# Resistance of Cereal-Husk-Reinforced PVC Terrace Profiles to *Agaricomycetes* Fungi

**DOI:** 10.3390/ma18122860

**Published:** 2025-06-17

**Authors:** Mariia Goron, Ewa Sudoł, Ewelina Kozikowska

**Affiliations:** Construction Materials Engineering Department, Instytut Techniki Budowlanej, 00-611 Warsaw, Poland; e.sudol@itb.pl (E.S.); e.kozikowska@itb.pl (E.K.)

**Keywords:** fungi, oat husks, millet husks, terrace profiles, performance

## Abstract

Plant-particle-reinforced polymer composite products are widely used in construction. New terrace profiles, reinforced with oat or millet husks, are being considered. However, their resistance to wood-decomposing Agaricomycetes fungi requires investigation due to their intended environmental exposure. Susceptibility to *Coniophora puteana*, *Coriolus versicolor*, and *Gleophyllum trabeum* was tested. Well-known rice-husk-reinforced profiles were used as the reference material for the analysis. The bioresistance of the oat-husk-reinforced profiles was similar to that of the reference profile. Minor mycelium development and changes in the surface morphology, mass, and flexural strength were found. Millet husk-reinforced profiles showed greater fungal susceptibility, questioning their suitability for the intended application. *Coniophora puteana* was the most aggressive among all the profiles.

## 1. Introduction

For many years, the terraces in residential houses and public buildings have typically been constructed from wood. They are often situated near the ground, close to plants, in shaded and humid areas. Wood is highly susceptible to microbiological degradation, particularly when exposed to high humidity and variable temperatures [1]. Furthermore, the fungi and bacteria naturally present on wooden surfaces degrade their appearance and, given suitable conditions, cause structural damage to wooden terrace boards [2].

**Figure 1:**
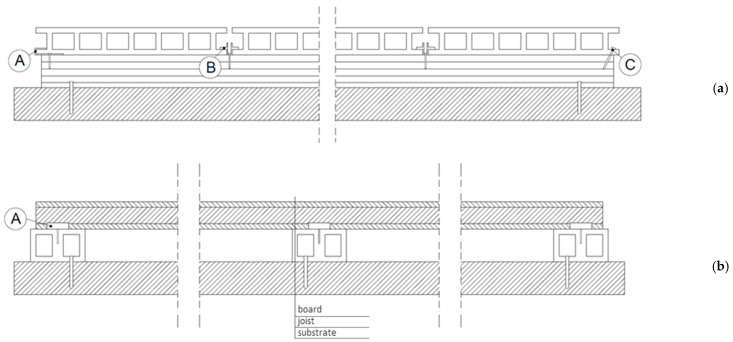
Sample cross-section of a terrace board (**a**) perpendicular to the board length, (**b**) parallel to the board length (A—starting clip, B—installation clip, C—drive screw) [8].

The dynamic development of polymer composites with lignocellulose particle fillers offers an alternative solution. Biocomposites initially found application in the automotive, aviation, and shipbuilding sectors [3,4]. Currently, they play a vital role in construction. Composite profiles are popular in façade facing, flooring (including terraces), promenades, piers, landscape architecture, window and door joinery, and interior design items [5,6,7]. Extruded closed profiles are typical for terraces. They are used as floor decks and joists (Figure 1).

Despite being designed to minimise water absorption, biocomposite profiles can biodegrade in high-humidity conditions, especially if they have a significant natural particle content [9].

Terrace board susceptibility to fungal growth depends on factors such as filler type and amount, material use, humidity, and temperature. The optimum air temperature for fungal growth ranges from 3 °C to 40 °C. Some fungi are resistant to low temperatures, but most form sclerotia to survive winter. High air humidity and the water access to the terrace can increase the moisture content of filler particles to 70%. It should be highlighted that *Agaricomycetes* fungi grow actively at a substrate moisture content of over 28% [9]. Furthermore, long-lasting exposure to water causes material swelling and the formation of microcracks, which facilitates the penetration of microorganisms [10]. Laboratory tests on wooden and polymer composites revealed that long-lasting exposure to moisture can deteriorate their performance by as much as 50% [11].

Water absorption capability can lead to susceptibility to fungal growth, depending on the composite composition and environmental conditions. Fungi, such as *Trametes versicolor* and *Coniophora puteana*, are the main contributors to wood and wood-like material biodegradation. In composite boards, fungal attack can lead to the gradual decomposition of the organic ingredients of the filler, compromising the material structure [12]. Wood and polymer-based materials can lose up to 50% of their mechanical characteristics when exposed to humidity and fungi [13,14,15].

Fungi may cause brown rot (e.g., *Coniophora puteana*), which involves the use of cellulose as the nutritional material, or white rot (e.g., *Coriolus versicolor*), which attacks lignin [16]. Fungi can act superficially or deeply, modifying the physical characteristics of the affected material, including mass loss and changes in colour and microstructure.

As heterotrophic organisms, fungi derive energy and nutrients from the decomposition of organic compounds. They achieve this by producing and releasing hydrolytic enzymes, which break down large chemical compounds (like polysaccharides, proteins, and lipids) into simpler forms for fungal absorption [17].

**Figure 2:**
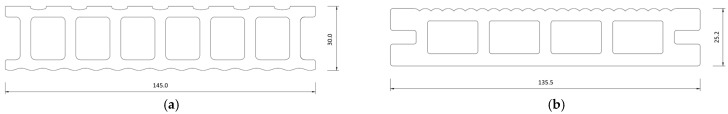
Sample shape of terrace boards (**a**) grooved on both sides, (**b**) grooved on one side [8].

It should be noted that the surface treatment methods of and geometries of terrace profiles promote the growth of microorganisms. The extruded profile surface is smooth, shiny, and sleek. To make it look like wood and hence improve its visual attractiveness and reduce the risk of slipping [18], usable surfaces are ground or brushed [19]. Machined boards are more susceptible to environmental factors than non-machined products owing to the exposure to lignocellulose particles [20,21]. The usable surfaces of the terrace boards are often milled. Grooving is performed on both sides with a diversified geometry or on only one side (Figure 2). Both sides of the profiles are usable. Water stays longer in the grooves than on flat surfaces, thereby promoting microorganism growth.

Polyvinyl chloride (PVC), polyethene of high density (PEHD), and polypropylene (PP) matrix profiles prevail during construction. Typical fillers include shredded wood waste [22,23], also called wood–plastic composites (WPCs), and pulverised rice husks [24,25]. There has been the development of composites with other cereal husks [26,27,28]. Since plant particles are lignocellulose structures whose chemical composition varies depending on the plant species, using new fillers may change the composite’s characteristics [29,30]. The new profiles’ fitness for construction should be assessed according to the rules valid for construction materials, following the usability criteria in reference to the collection of key features for specific use [31]. Microbiological resistance is among the key aspects for assessing the fitness of terrace profiles for construction, considering the conditions of their use.

This study assessed the susceptibility of new terrace profile solutions based on shredded oats and millet husks as reinforcements. The trend towards managing grain waste is part of the wider trend of recycling waste for use in the construction industry [32]. The use of residual materials from the hulling of oat and millet grains in the production of polymer composites is an environmental benefit. Their susceptibility to *Coniophora puteana*, *Coriolus versicolor*, and *Gloophyllum trabeum* of the *Agaricomycetes* type was analysed for the first time. The reference fungi are common in environments typical of terrace conditions.

**Figure 3:**
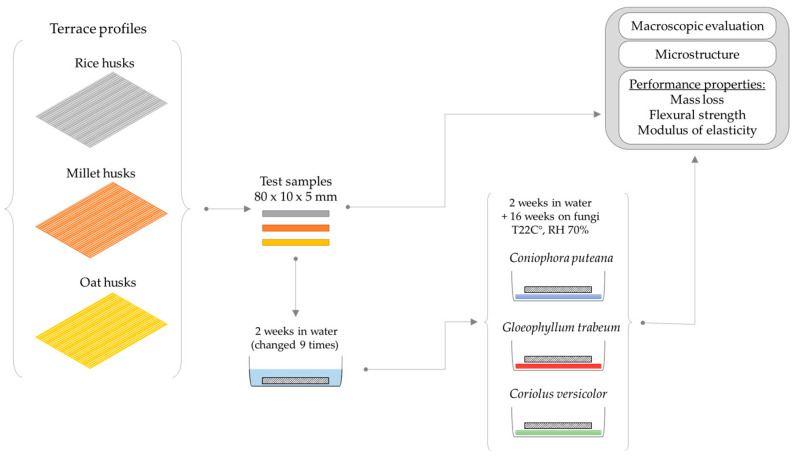
Workflow diagram.

After 16 weeks of exposure, aspects such as mycelium growth on the specimen surfaces, changes in the usable surface morphology based on scanning microscope images, mass loss, and the decrease in the flexural strength of the terrace profiles were evaluated. The analyses were carried out with reference to rice-husk-reinforced profiles, which are popular in construction and terrace flooring. The workflow is illustrated in Figure 3.

## 2. Materials and Methods

### 2.1. Test Specimens

Three types of composite profiles with a PVC matrix, and pulverised rice, oats, and millet husks were tested (Table 1). These profiles were produced industrially via extrusion. The usable surfaces were subjected to brushing, which is the standard for construction profiles. The profiles were not milled.

Specimens sized 80 mm × 10 mm × 5 mm were cut from the profiles and exposed to ageing in a distilled water bath (full immersion) for two weeks; the water was changed nine times during that period [33]. A control series was also prepared; it included specimens seasoned under laboratory conditions.

### 2.2. Fungal Exposure

Microbiological tests comprised several stages, including preparation of fungi sub-cultures, inoculation of spores on the medium, and adequate exposure of test specimens.

*Coniophora puteana* (Schumacher ex Fries) BAM Ebw. 15, *Gloeophyllum trabeum* (Persoon ex Fries) BAM Ebw. 109 and *Coriolus versicolor* (also *Trametes versicolor*) (Linnaeus) subcultures were inoculated from the stock cultures. The fungi were inoculated into test tubes on an agar–malt medium composed of 40 g malt extract, 35 g agar, and 1000 mL of water pre-sterilised in an autoclave at 121 °C and 1 atm pressure. They were cultured in a culture chamber at 22 ± 1 °C and 70 ± 5% RH for four weeks.

The media for long-term exposure to fungi were prepared in sterile Kolle flasks using a sterile medium same as for subcultures. *Coniophora puteana*, *Coriolus versicolor*, and *Gleophyllum trabeum* were inoculated on the medium in Kolle flasks using hyphae from subcultures. The flasks were incubated in a culture chamber at 22 ± 1 °C and 70 ± 5% RH until the mycelium covered the entire surface of the medium.

All specimens prepared according to Section 2.1 were exposed to fungi. The specimens were placed on the mycelium so that the usable (brushed) surface directly touched it. The specimens were then moved to the culture chamber for 16 weeks and maintained at 22 ± 1 °C and 70 ± 5% RH.

### 2.3. Macroscopic Analysis

Profile specimens were macroscopically evaluated after exposure to the fungi. They were removed from the culture flasks with pliers, without compromising the mycelium structure. The degree of mycelium coverage was visually assessed during daylight. The mycelium covering the entire surface of the specimen and visible in the photographs was considered well developed. Images were captured using a Canon SX30 IS 14 Mpix camera (Canon Europe, Amstelveen, The Netherlands) against a light background.

### 2.4. SEM Analysis

The microstructure of the usable surface of the profiles was analysed before and after exposure to *Coniophora puteana*, *Coriolus versicolor*, and *Gloophyllum trabeum*. A Sigma 500 VP scanning electron microscope (Carl Zeiss Microscopy GmbH, Köln, Germany) with cold-field emission was used. Specimens for which visual assessment revealed the highest mycelial growth were selected for microstructure observation after exposure to fungi. Immediately before the test, the mycelia on the specimens were cleaned with pressurised water and dried for seven days at 40 ± 2 °C. Tests were performed on gold-sprayed specimens using an SE detector (Carl Zeiss Microscopy GmbH, Köln, Germany) at an accelerating voltage of 5 keV. All observations were made at 200× magnification.

### 2.5. Performance

The mass loss and changes in the flexural strength after exposure to fungi were also investigated.

The mass change was evaluated using the initial mass of the specimens and the mass of the dry specimens cleaned of mycelium after exposure to fungi as the final data. The cleaned specimens were dried to a constant mass value to determine mass loss. The total mass loss was calculated according to Formula (1). The results are expressed as percentages.

(1) $$\Delta m_{d}=\frac{m_{i}-m_{c}}{m_{c}}\cdot 100$$

where *m_i_* is the specimen’s initial dry mass, in grams; *m_c_* is the dry specimen’s mass after exposure to microorganisms, in grams.

The initial dry mass of the specimens was calculated from their initial aged mass and the material’s moisture content coefficient (derived from Formula (2)), as pre-exposure drying might increase microorganism susceptibility. A separate dried specimen series was used to calculate the coefficient.

(2) $$F_{i}=1-\frac{m_{w}-m_{d}}{m_{w}}\cdot 100$$

where *m_w_* is the aged specimen’s initial mass, in grams; *m_d_* is the mass of the specimen dried after ageing, in grams.

The flexural strength and modulus of elasticity of the specimens before and after exposure to fungi were tested according to ISO 178 [34]. The test was conducted using a Class 1 strength-testing machine (Instron, Darmstadt, Germany). The specimens were placed on 5 mm radius supports spaced every 64 mm, with the brushed side facing downward. Thus, tensile stress occurred on the surface exposed to fungi. They were exposed to the force transmitted by a 5 mm radius loading piece, applied in the middle of the support spacing. A load of 2 mm/min was applied until destruction occurred. An analogous testing method was previously used to evaluate the impact of microorganisms on the performance of polymer composite construction products [35]. The maximum force was recorded, and its value was used to calculate the flexural strength according to (3) [34].

(3) $$\sigma =\frac{3FL}{2bh^{2}}$$

where *F* is the maximum force, in N; *L* is the support spacing, in mm; *b* is the width, in mm; *h* is the thickness, in mm.

A load-deflection curve was recorded during bending in a linearly elastic range, including the force and deflection values corresponding to strains of ε*_f_*_1_ = 0.0005 and ε*_f_*_2_ = 0.0025. The deflection values of *f*_1_ and *f*_2_ were calculated using Equation (4) [34].

(4) $$f_{1}=\frac{\varepsilon_{f1}L^{2}}{6h};\;f_{2}=\frac{\varepsilon_{f2}L^{2}}{6h}$$

where *L* is the support spacing, in mm; *h* is the thickness, in mm.

The force values were recorded when the ε*_f_*_1_ and ε*_f_*_1_ strains were used to determine the values of the σ*_f_*_1_ and σ*_f_*_2_ normal stresses. The *E* modulus was calculated according to Equation (5) and is expressed in MPa [34].

(5) $$E=\frac{\sigma_{f2}-\sigma_{f1}}{\varepsilon_{f2}-{\;\varepsilon }_{f1}}s$$

where σ*_f_*_1_, σ*_f_*_2_ are the maximum normal stresses corresponding to the *f*_1_ and *f*_2_ stresses determined according to (4).

## 3. Results and Discussion

### 3.1. Macroscopic Analysis

**Figure 4:**
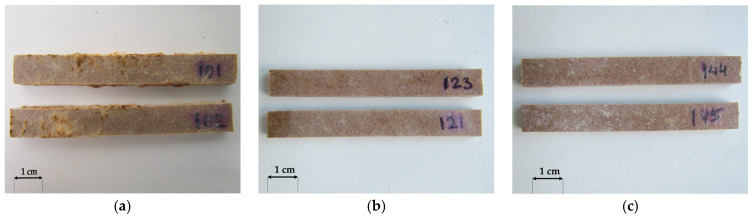
General view of selected rice-husk-reinforced composites after exposure to (**a**) *Coniophora puteana*, (**b**) *Gloeophyllum trabeum*, (**c**) *Coriolus versicolor*.

**Figure 5:**
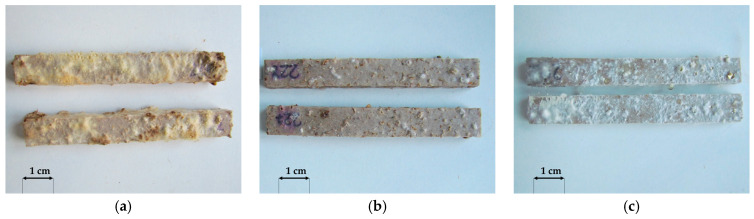
General view of selected millet-husk-reinforced composites after exposure to (**a**) *Coniophora puteana*, (**b**) *Gloeophyllum trabeum*, (**c**) *Coriolus versicolor*.

**Figure 6:**
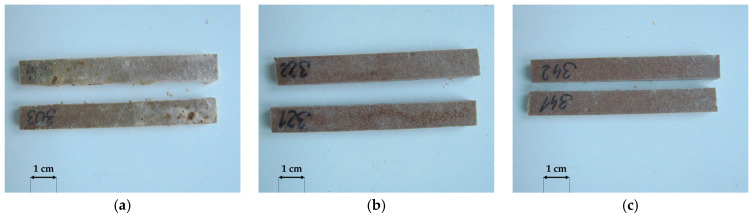
General view of selected oat-husk-reinforced composites after exposure to (**a**) *Coniophora puteana*, (**b**) *Gloeophyllum trabeum*, (**c**) *Coriolus versicolor*.

A macroscopic assessment of the terrace profile specimens after 16 weeks of exposure to *Agaricomycetes* fungi was used to analyse the susceptibility of the profiles to fungal growth. *Coniophora puteana* demonstrated the highest mycelial activity among all strains tested. It developed on all tested products (Figure 4a, Figure 5a and Figure 6a). The largest mycelium formed on specimens containing millet husks (Figure 4a), and the smallest mycelium formed on specimens with oat husks (Figure 6a). According to previous studies, *C. puteana* is highly active in polymer composites with natural fillers [36]. *Gloeophyllum trabeum* mycelium was the least active (Figure 4b, Figure 5b and Figure 6b), but it should be emphasised that this fungus is rarely aggressive [37]. For specimens containing rice husks, the growth of *Coriolus versicolor* mycelia was insignificant (Figure 4c). However, *C. versicolor* grew intensively on the specimens with millet husks and covered a considerable part of the specimen surface (Figure 5c). No visible traces of *C. versicolor* (Figure 6c) were observed in the millet-husk-reinforced specimens.

The results revealed the lower susceptibility of oat husk-reinforced profiles to fungal growth and outclassed the rice-husk-reinforced reference profile. Millet-husk-reinforced profiles demonstrated high sensitivity to biodegradation organisms.

### 3.2. Microstructure Analysis

Morphologically, we qualitatively assessed damage to the profiles’ usable surface before and after exposure to *Coniophora puteana*, *Gloophyllum trabeum*, *and Coriolus versicolor*.

**Figure 7:**
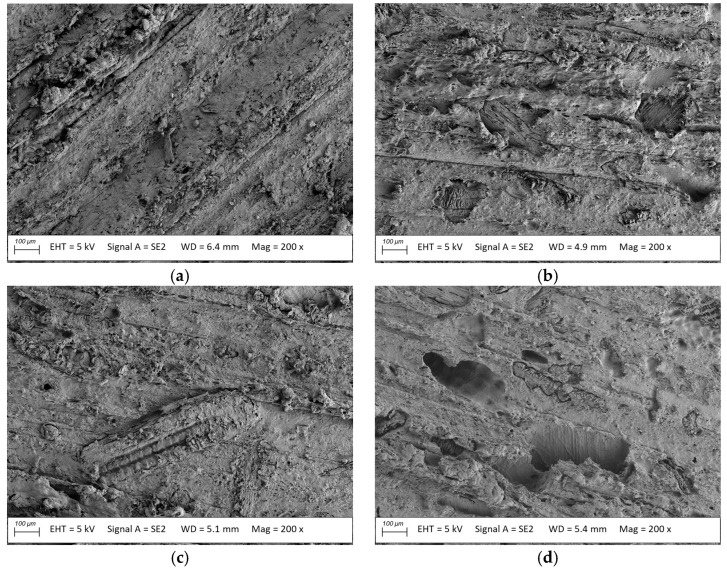
Microstructure of the usable surface of rice-enriched profiles (**a**) before and after exposure to (**b**) *Coriolus versicolor*, (**c**) *Gleophyllum trabeum*, (**d**) *Coniophora puteana*; magnification: 200×.

**Figure 8:**
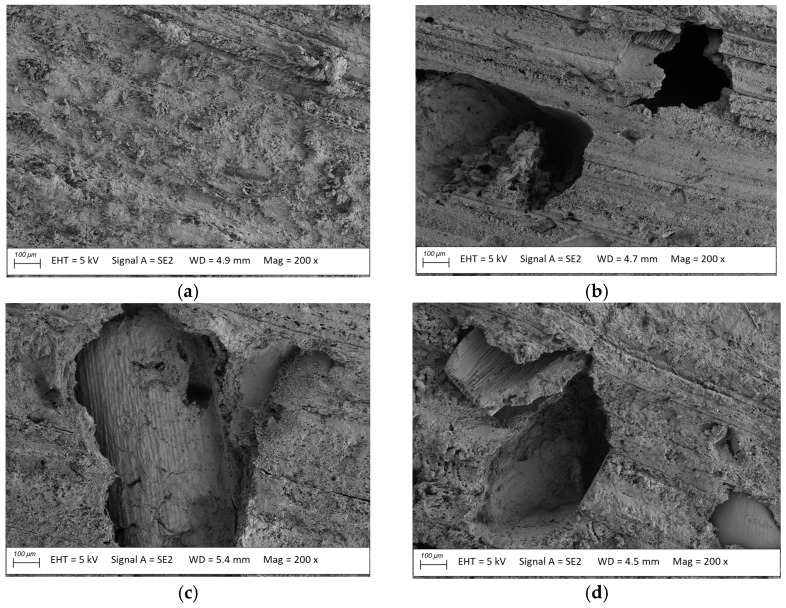
Microstructure of the usable surface of millet-enriched profiles (**a**) before and after exposure to (**b**) *Coriolus versicolor*, (**c**) *Gleophyllum trabeum*, (**d**) *Coniophora puteana*; magnification: 200×.

**Figure 9:**
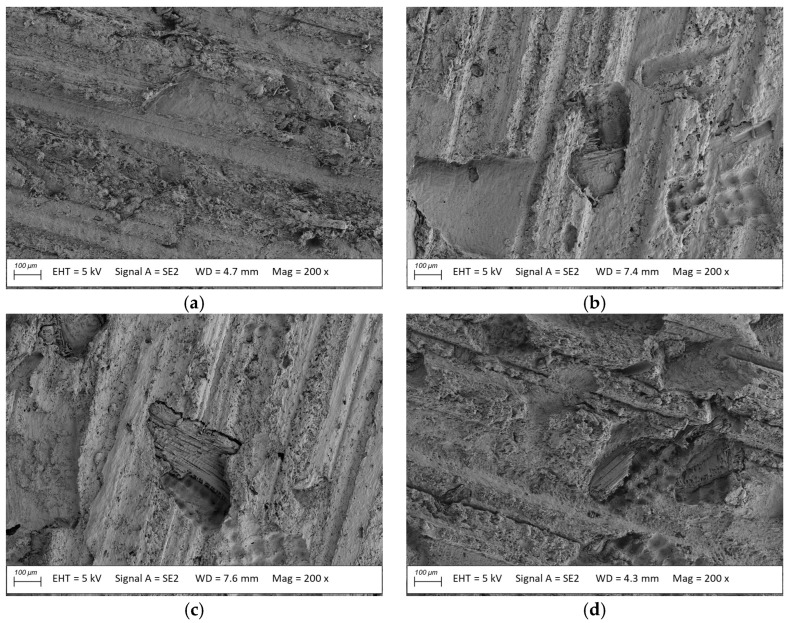
Microstructure of the usable surface of oat-enriched profiles (**a**) before and after exposure to (**b**) *Coriolus versicolor*, (**c**) *Gleophyllum trabeum*, (**d**) *Coniophora puteana*; magnification: 200×.

The surface microstructures before the exposure of the rice-, oat-, and millet-husk-reinforced profiles revealed that the filler particles were uniformly coated with the polymer. No visible filler clusters or exposed lignocellulose particles were observed in the images. Specifically oriented streaks and scratches characteristic of a brushed surface topography were observed on the surface of the profiles (Figure 7a, Figure 8a and Figure 9a). Microscopic images of the surface of the rice-husk-reinforced profiles after exposure to *Coriolus versicolor* and *Gleophyllum trabeum* (Figure 7b,c) were similar to those observed for the material before exposure. No changes were observed after exposure to any of the fungal species. The exposed filler fragments (Figure 7b,c) likely resulted from the humid conditions during the exposure to the mycelium. The polymer matrix could have lost its integrity, even though no traces of the exposed filler were observed before exposure. Lignocellulose particles are strongly hydrophilic and swell easily [5,38], which may damage the surrounding polymer matrix. The results revealed no destructive impact of *Coriolus versicolor* or *Gleophyllum trabeum* on the rice-husk-reinforced profiles. After exposure to *Coniophora puteana*, voids that could attributed to the loss of filler particles or their fragments were observed. This indicated that decomposition occurred after exposure to fungi [39].

Images of the millet-husk-reinforced profiles’ microstructure revealed voids ranging in size from 500 to 1000 µm after exposure to *Coriolus versicolor*, *Gleophyllum trabeum*, and *Coniophora puteana* (Figure 8b–d). They demonstrated the filler loss in the polymer matrix, which resulted either from filler sedimentation during ageing in a water bath for two weeks [38] or degradation after exposure to the fungi. Simultaneously, the images of the profile microstructure after the exposure to *Gleophyllum trabeum* (Figure 8c) showed a partial loss of the filler fragment after exposure to fungi, confirming partial biodegradation of the material [39,40]. The filler loss from the polymer matrix and its significant degradation may have resulted from the low homogeneity of the millet husk particles in the polymer matrix.

No voids were evident in the microstructure images of the oat-husk-reinforced profiles after exposure to *Coriolus versicolor*, *Gloeophyllum trabeum*, and *Coniophora puteana* (Figure 9b–d), suggesting low fungal susceptibility. The exposed filler fragments observed in the images were likely caused by the humid environment and the beginning of fungal degradation. The filler particles were apparent but not severely damaged (Figure 9b–d), which confirmed partial biodegradation. The microscopy image of the oat-husk-reinforced composite surface was similar to that of the reference material, that is, a rice-husk-reinforced composite.

### 3.3. Performance

**Figure 10:**
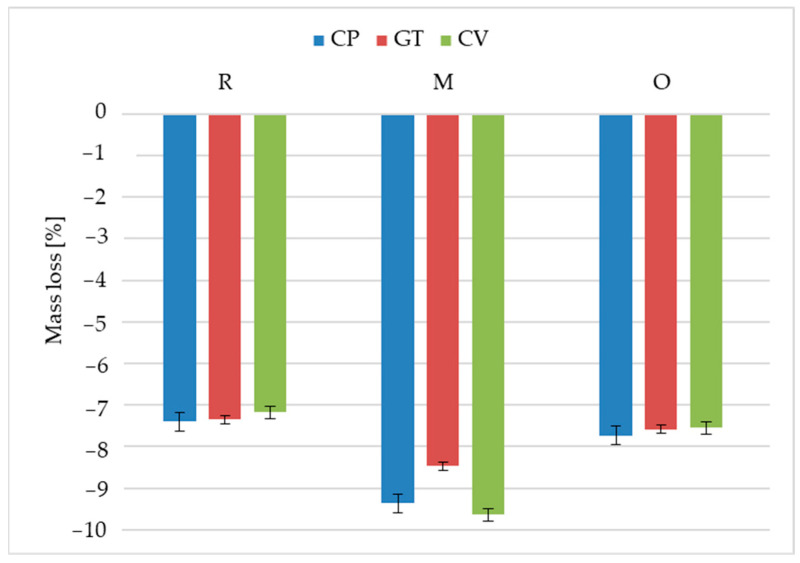
Test results for mass loss for composites reinforced with rice husks (R), millet husks (M), and oat husks (O) after exposure to *Coriolus versicolor* (CV), *Gloeophyllum trabeum* (GT), and *Coniophora puteana* (CP) fungal strains. The error bars illustrate the standard deviation.

The analysis of the mass loss data (Figure 10, Table 2) after exposure to the fungi revealed that the rice-husk-reinforced reference material was the most resistant to mass changes. The reported mass loss was slightly higher than 7%, regardless of the fungi used. It corresponded to the microstructure tests, where no significant changes were observed after *Coriolus versicolor* or *Gloeophyllum trabeum* exposure. The fungi only caused the exposure of filler fragments, as reported in other studies [41,42]. Exposure to *Coniophora puteana* caused voids of filler loss, also observed in the microscopy images. However, this did not result in a more significant mass loss, which amounted to 7.4%. The empty pores might have been filled with mycelium during the mass change tests, which might have been removed when the specimens were cleaned for microstructure observation.

The most considerable mass loss, up to 9.6%, was found for the millet-husk-reinforced profile after exposure to *Coriolus versicolor*. Surface microstructure analysis revealed numerous voids of significant sizes, ranging from 500 to 1000 µm (Figure 8), suggesting filler loss in the polymer matrix. Millet husk particles larger than oat or rice husks improve the nutrient availability for microorganisms, and large voids facilitate fungal migration into the material structure [43].

The oat-husk-reinforced profiles demonstrated mass loss levels similar to those of the reference material, regardless of the fungal type, ranging from 7.6% to 7.7%. The microstructural analysis did not reveal large voids in the polymer matrix. Minor reinforcement losses were found, confirming the low susceptibility of the material to biodegradation.

**Figure 11:**
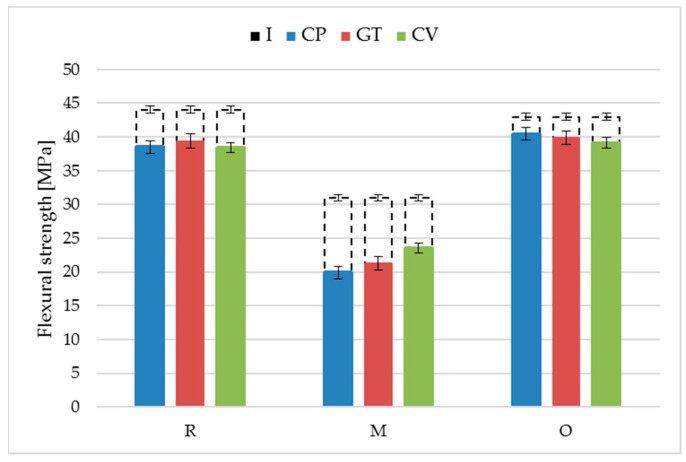
Test results of flexural strength for composites reinforced with rice husks (R), millet husks (M), and oat husks (O) before (I) and after exposure to *Coriolus versicolor* (CV), *Gloeophyllum trabeum* (GT) and *Coniophora puteana* (CP) fungal strains. The error bars illustrate the standard deviation.

**Figure 12:**
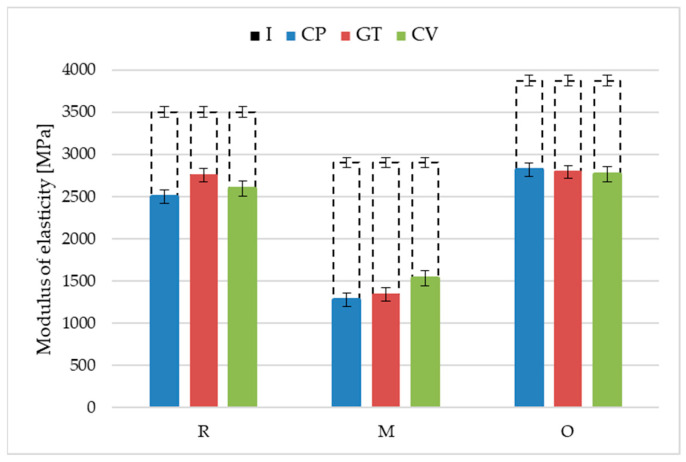
Test results for the modulus of elasticity for the composites reinforced with rice husks (R), millet husks (M), and oat husks (O) before (I) and after exposure to *Coriolus versicolor* (CV), *Gloeophyllum trabeum* (GT) and *Coniophora puteana* (CP) fungal strains. The error bars illustrate the standard deviation.

The highest flexural strength (44.1 MPa) and modulus of elasticity (3490 MPa) were obtained for the reference rice-husk-reinforced profiles before exposure (Figure 11 and Figure 12, Table 3 and Table 4).

Exposure to lignocellulose-decomposing fungi deteriorated the flexural strength of the rice-husk-reinforced profiles. Similar changes were found for all three fungi: *Coniophora puteana*, *Gloeophyllum trabeum*, and *Coriolus versicolor*, where the strength decreased to 38.5 MPa, 39.4 MPa, and 38.4 MPa, respectively. The minor but uniform strength decrease meant that the fungal growth mechanism was ineffective in degrading husk particles; the humid environment could have been the biodegradation trigger [44,45]. The value of the modulus of elasticity after exposure to *Coniophora puteana*, *Gloeophyllum trabeum*. and *Coriolus versicolor* was more diversified—the obtained values amounted to 2500 MPa, 2750 MPa, and 2590 MPa. The lowest value was obtained for specimens exposed to *Coniophora puteana*. This was the only of the three tested fungi that caused the partial decomposition of the rice husk particles on the surface of the profiles (Figure 7). The observed changes progressed differently from those in wood, where fungal exposure reduced the modulus of elasticity to below the strength [46]. It should be noted that the mycelium penetration in the composite structure was not as significant as that in wood.

The strength tests on the millet-husk-reinforced profiles before exposure revealed that their flexural strength (30.8 MPa) and modulus of elasticity (2910 MPa) values were much lower than those of the reference material. This was related to the differences in the filler particle shapes and sizes [4]. Exposure to fungi for 16 weeks aggravated this difference. The three fungi exerted similar impacts on the millet-husk-reinforced profiles, reducing their flexural strength to 20.0 MPa (*C. puteana*), 21.3 MPa (*G. trabeum*), and 23.5 MPa (*C. versicolor*), and the modulus of elasticity to 1270 MPa (*C. puteana*), 1340 MPa (*G. trabeum*) and 1530 MPa (*C. versicolor*). The surface morphology analysis revealed that the millet husk particles were larger than the others, which promotes water absorption, and consequently, swelling and adhesion deterioration at the phase border [22]. The microscopy images revealed significant surface degradation. It was hypothesised that the microorganisms used the millet husk particles as a nutritional material, leaving voids of considerable size in the polymer matrix. Bending was performed such that the surface exposed to the fungi was stretched. The composite degradation in the tension zone reduced the flexural strength. This also led to higher deformability, as evidenced by the reduced modulus of elasticity. The susceptibility of the millet husks to humidity levels being higher than those of other cereals should not be neglected [22,37,47].

The flexural strength and modulus of elasticity of the oat-husk-reinforced terrace profile specimens before exposure were similar to those of the reference product. They amounted to 43.4 MPa and 3870 MPa, respectively. The obtained strength parameters, comparable to those of the reference material, suggested the correct preparation of the oat husk particles and adequate dispersion in the polymer matrix [26]. Long-term exposure to fungi slightly deteriorated these parameters. A decrease in the flexural strength was found, amounting to 40.4 MPa (*C. puteana*), 39.9 MPa (*G. trabeum*), and 39.2 MPa (*C. versicolor*); the same was true for the modulus of elasticity to 2820 MPa (*C. puteana*), 2800 MPa (*G. trabeum*), and 2770 MPa (*C. versicolor*). The strength changes in the millet-husk-reinforced profiles were similar to those of the rice-husk-reinforced profiles, suggesting a lack of easily available nutrition for microorganisms. This was confirmed by microscopy observation. Despite the exposure of the particles, there were no voids, and only minor traces of fungi influenced the natural particles. The results suggest a high resistance of the oat-husk-reinforced profiles to biodegradation [48,49].

The microbiological susceptibility of the cereal husk-reinforced profiles varied depending on the fungal species. *C. puteana* caused the most considerable declines in the flexural strength and modulus of elasticity. The species is characterised by aggressive action on materials, which are the nutritional medium for the mycelium [50]. Its degradation mechanism is primarily based on the selective decomposition of polysaccharides, leading to a loss of the structural integrity of the material and deterioration of its mechanical properties [51]. The process is more intensive under high-humidity conditions because the fungus effectively develops its mycelium in the material’s pores, increasing water retention and accelerating degradation. A specific feature of *Coniophora puteana* is the mycelium’s high moisture content; after development, it keeps the environment and the covered surface humid [52]. Under such conditions, the terraces are more susceptible to degradation.

*Gloeophyllum trabeum* acts similarly to *Coniophora puteana*, but the mycelium develops at a slower rate, so its influence on the mechanical properties of the profiles can increase with time [53]. *Gloeophyllum trabeum* and *Coniophora puteana* are brown rot fungi that degrade cellulose and hemicellulose, leaving lignin as the crumbling matrix. Small empty pores containing natural structural residues formed in composites with smaller particles after exposure to brown rot fungi [54,55]. For larger particles, the pores formed can be too large to maintain coherence, and they are easily destroyed after loading, as was the case with the millet-husk-reinforced profiles.

*Coriolus versicolor*, a white rot fungi, decomposes lignin, leading to fairly uniform structural deterioration and a moderate strength decrease [42,56]. If the composite polymer matrix degraded and plant particles lacked fungal protection, the profile strength decline surpassed that of the oat-husk-reinforced profiles.

## 4. Conclusions

The following conclusions can be drawn based on the analysis of the results:

- The resistance of oat-husk-reinforced profiles to wood-decomposing fungi was similar to that of the reference rice-husk-reinforced profiles. A similar growth rate of *Coniophora puteana*, *Gloeophyllum trabeum* and *Coriolus versicolor* was found for both solutions. The changes in the surface morphology were also identical. The mass loss and decreases in flexural strength and modulus of elasticity after exposure to the fungi were similar.
- The millet-husk-reinforced profiles were susceptible to *Agaricomycetes* fungi. The mycelia were more developed, especially in the case of *Coniophora puteana* and *Coriolus versicolor*. Surface degradation was observed, including numerous voids left after the fillers, contributing to weight, flexural strength, and modulus of elasticity changes.
- Considering the biodegradation resistance of the oat-husk-reinforced profiles, they may be suitable for use in terraces. This may be a reason for further considering the use of residual materials from the hulling of oat grains in the production of polymer composites, which is an environmental benefit. The susceptibility of millet-husk-reinforced profiles to microbiological factors seems too high to be appropriate for such applications.
- Various fungal species had different effects on cereal-husk-reinforced profiles. *Coniophora puteana* and *Coriolus versicolor* affected the performance of the composites. Further studies are planned to determine the susceptibility of cereal-husk-reinforced profiles to other microorganism groups and environmental factors. This project was undertaken with the main goal of evaluating the changes in the surface properties of terrace profiles under abiotic factors and the effect of these changes on the susceptibility to fungal and algal growth. The influence of abiotic factors was simulated by artificial methods, as well as several years of observations under natural conditions. The new experimental data will be analysed in terms of the service life of terrace profiles.

## Figures and Tables

**Table 1:** The composition of the composites.

Profile	Plant Filler	Matrix	Mineral Filler
O	pulverised oat husks (30 phr)	PVC (100 phr)	CaCO_3_ (50 phr)
M	pulverised millet husks (30 phr)		
R	rice husks (details no available)		no data available

**Table 2:** Statistical analysis of the test results for the mass loss for composites reinforced with rice husks (R), millet husks (M), and oat husks (O) and after exposure to fungi.

Profile	Fungi	Min	Max	Medium	Median
R	*Coniophora puteana*	−7.49	−7.23	−7.40	−7.44
	*Gloeophyllum trabeum*	−7.56	−7.19	−7.35	−7.31
	*Coriolus versicolor*	−7.42	−6.98	−7.17	−7.13
M	*Coniophora puteana*	−10.07	−8.43	−9.37	−9.47
	*Gloeophyllum trabeum*	−9.20	−8.08	−8.47	−8.39
	*Coriolus versicolor*	−10.42	−8.75	−9.64	−9.74
O	*Coniophora puteana*	−7.98	−7.55	−7.73	−7.65
	*Gloeophyllum trabeum*	−7.84	−7.35	−7.58	−7.57
	*Coriolus versicolor*	−7.80	−7.44	−7.55	−7.48

**Table 3:** Statistical analysis of test results for flexural strength for composites reinforced with rice husks (R), millet husks (M), and oat husks (O) and after exposure to fungi.

Profile	Fungi	Min	Max	Medium	Median
R	*Coniophora puteana*	36.63	40.72	38.50	38.26
	*Gloeophyllum trabeum*	37.03	40.82	39.41	39.53
	*Coriolus versicolor*	36.61	40.68	38.42	38.45
M	*Coniophora puteana*	15.54	25.17	19.95	18.52
	*Gloeophyllum trabeum*	19.34	22.95	21.25	21.46
	*Coriolus versicolor*	20.78	27.88	23.51	23.11
O	*Coniophora puteana*	39.46	42.04	40.48	40.23
	*Gloeophyllum trabeum*	36.63	42.63	39.83	39.96
	*Coriolus versicolor*	36.42	40.40	39.15	39.79

**Table 4:** The statistical analysis of the test results for the modulus of elasticity for the composites reinforced with rice husks (R), millet husks (M), and oat husks (O) and after exposure to fungi.

Profile	Fungi	Min	Max	Medium	Median
R	*Coniophora puteana*	2288	2884	2495	2424
	*Gloeophyllum trabeum*	2434	2910	2749	2784
	*Coriolus versicolor*	2276	2786	2594	2605
M	*Coniophora puteana*	1085	1515	1274	1217
	*Gloeophyllum trabeum*	1119	1522	1337	1352
	*Coriolus versicolor*	1204	1695	1529	1619
O	*Coniophora puteana*	2611	2923	2819	2846
	*Gloeophyllum trabeum*	2521	3031	2791	2811
	*Coriolus versicolor*	2473	3089	2766	2747

## Data Availability

The original contributions presented in this study are included in the article. Further inquiries can be directed to the corresponding author.
